# A Puzzle of Hemolytic Anemia, Iron and Vitamin B12 Deficiencies in a 52-Year-Old Male

**DOI:** 10.1155/2013/708489

**Published:** 2013-09-05

**Authors:** Suartcha Prueksaritanond, Aram Barbaryan, Aibek E. Mirrakhimov, Palacci Liana, Alaa M. Ali, Alan D. Gilman

**Affiliations:** Saint Joseph Hospital, Department of Internal Medicine, University of Illinois at Chicago, 2900 N., Lake Shore, Chicago, IL 60657, USA

## Abstract

A 52-year-old male with no significant past medical history reports increasing generalized fatigue and weakness for the past 2 weeks. Physical examination reveals jaundice and pallor without organomegaly or lymphadenopathy. His hemoglobin was 5.9 g/dL with a mean corpuscular volume of 87.1 fL and elevated red blood cell distribution width of 30.7%. His liver function test was normal except for elevated total bilirubin of 3.7 mg/dL. Serum LDH was 701 IU/L, and serum haptoglobin was undetectable. Further investigation revealed serum vitamin B12 of <30 pg/mL with elevated methylmalonic acid and homocysteine level. In addition, serum ferritin and transferrin saturation were low. The patient was diagnosed with hemolytic anemia secondary to vitamin B12 deficiency with concomitant iron deficiency anemia.

## 1. Introduction

Vitamin B12 (also known as cobalamin) deficiency can cause reversible bone marrow failure and demyelinating disease due to its inherent function in erythropoiesis and myelination of the central nervous system [1]. Any conditions that cause malabsorption of vitamin B12 as well as dietary deficiency can lead to cobalamin deficiency. Pernicious anemia is the most common cause of vitamin B12 deficiency [1]. Below we present a case of vitamin B12 deficiency due to nutritional deficiency with coexisting iron deficiency anemia.

## 2. Case Presentation

A 52-year-old male was initially presented in an ambulatory clinic complaining of fatigue and weakness for 2 weeks. The patient also complained of frequent epistaxis secondary to nose picking for 1 month. His fatigue was accompanied by dyspnea on exertion and lightheadedness which have increased in frequency in the last 4-5 days prior to the presentation. He denied similar symptoms in the past. He reported poor appetite but no weight loss or strange craving. Other than the symptoms reported previously, the review of symptoms was negative, including neurological complaints. Complete blood count (CBC) was taken in the clinic and he was found to have hemoglobin (Hb) of 6.2 g/dL. The patient was subsequently admitted to the hospital for further workup.

Further history revealed recent upper respiratory tract infection 1 month prior to the admission. The patient's symptoms at that time consisted of sore throat, runny nose, and low grade fever. The symptoms resolved on thier own after 5 days. There was no rash or joint pain related to the recent upper respiratory tract infection. He denied any history of bleeding disorder or any past medical history including blood transfusion. The only medication reported was Metamucil to relieve occasional constipation. 

The patient was originally from Mexico. He has been living in the United Stated for the last 14 years and has not recently visited his home country. He is married and has 2 children, age 16 and 14 years, which are healthy. The patient reported that his sister and his niece may have had history of anemia but he does not know the diagnosis. He denied any history of tobacco or drug use. He admitted to drink alcohol about 6 beers per week. He works in a pastry shop as a box assembler.

On admission, the patient was alert, oriented, and not in any distress. Physically, he looked thin and pale. Jaundice was also noted. His vital signs were blood pressure 107/59 mmHg, pulse 76/min, temperature 98.9 F, respiratory rate 18 min, oxygen saturation 100% on room air, height 165 cm, and weight 56 kg. His cardiopulmonary examination was normal. There was no lymphadenopathy. His abdomen was soft and nontender, with no organomegaly. There was no apparent rash, skin lesion, or joint swelling. Neurological exam was unremarkable. Rectal examination revealed normal prostate and no mass palpable. Brown stool was observed and bedside hemoccult test was negative.

**Figure 1:**
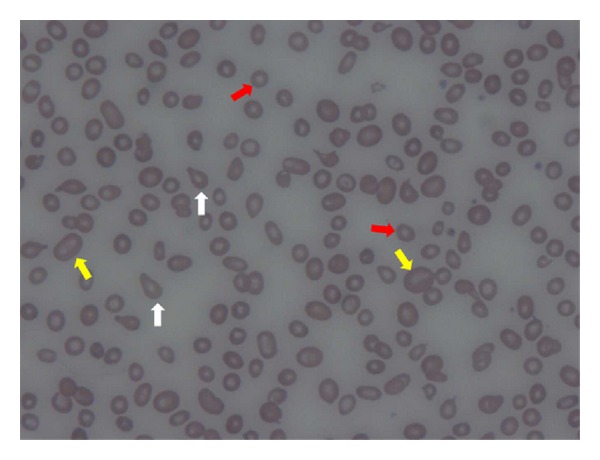
Examination of peripheral smear on admission showed marked anisocytosis and poikilocytosis. Microcytosis (red arrows) was predominant with interspersed large cells (yellow arrows) noted. Tear drop cells (white arrows), elliptocytes, and multiple fragmented red blood cells were also noted.

Repeat CBC revealed Hb of 5.9 g/dL and hematocrit (Hct) of 18.6% with normal white blood cell (WBC) and platelet count. Red cell indices were normal except for red blood cell distribution width (RDW) which was abnormally high. Review of peripheral blood smear (Figure 1) showed marked anisocytosis and poikilocytosis. Microcytosis was predominant with few large cells noted as well as tear drop cells and elliptocytes. Multiple fragmented red blood cells were also noted. WBC appeared normal. Platelet appeared low with occasional clumping. Complete metabolic panel showed elevated total bilirubin of 3.7 mg/dL with predominant indirect bilirubin level. Other values were normal. Reticulocyte count was 2.4%, but reticulocyte index was 0.4. Summary of laboratory investigation is presented in Table 1. 

Based on initial results, additional workup was sent including lactate dehydrogenase (LDH), haptoglobin, Coombs test, iron study, serum B12, serum folic acid, cold agglutinin, fibrinogen level, homocysteine level, methylmalonic acid (MMA) level, glucose-6-phosphate dehydrogenase level, chest radiograph, urine analysis, anti-nuclear antibody level, anti-phospholipid antibodies, ADAMTS-13 level, CD55/59, mycoplasma titer, and viral titer including HIV, viral hepatitis panel, EBV, CMV, and parvovirus. All results are summarized in Table 1.

Elevated LDH and low haptoglobin level were consistent with hemolytic anemia. Other test results were negative except for low serum vitamin B12 and abnormal iron study which was consistent with iron deficiency anemia. Serum homocysteine and methylmalonic acid were elevated which also confirmed the presence of vitamin B12 deficiency as the cause of hemolytic anemia. 

Once diagnosed, the patient was started on vitamin B12 supplement, 1000 mcg intramuscular injection daily, folic acid 1 mg daily, and ferrous sulfate 325 mg three times daily on hospital day 3. He also received 2 units of packed red cell transfusion prior to discharge. The patient was discharged on hospital day 4. Prior to transfusion, celiac disease antibodies and intrinsic factor antibody were also sent to further elucidate the cause of vitamin B12 deficiency. However, all antibodies were negative. Esophagogastroduodenoscopy (EGD) was not performed because the patient declined. His nutrition was later assessed after the diagnosis of vitamin B12 and iron deficiency and revealed that his diet was mostly consistent with vegetables and legumes. He does not eat a lot of meat, although he may have fish occasionally.

Upon followup, 5 days after discharge, the patient reported improvement of his symptoms. His Hb was at 9.3 g/dL and hematocrit of 29.7%. Hemolysis was improving with total bilirubin and LDH, and haptoglobin level normalized. Vitamin B12 injection was then reduced to once weekly, and it was later converted to oral form. Subsequent visits showed resolution of all symptoms with improvement of CBC, iron study, and serum B12 level.

## 3. Discussion

Anemia can be categorized into three pathophysiologic states: (1) blood loss, (2) defective erythropoiesis, and (3) destruction of erythrocytes [2]. Symptoms of anemia are nonspecific and may include tachycardia, dyspnea on exertion, pallor of nails and conjunctivae, fatigue, and decreased exercise tolerance.

Our patient presented with symptoms of increasing fatigue and weakness associated with dyspnea on exertion with extremely low Hb which confirmed the presence of anemia. Coexisting jaundice with elevated direct bilirubin and LDH, low haptoglobin level, and multiple fragmented red blood cell noted on peripheral smear indicate erythrocyte destruction or hemolytic anemia as the cause.

Hemolytic anemia represents a diverse group of diseases which can be divided in to congenital or acquired. Since the patient did not have history of anemia in the past, and no history of transfusion, no previous symptoms consistent with anemia or gallstone, no evidence of hepatosplenomegaly, it is unlikely that this is due to inherited conditions despite having suspected family history of anemia. Other confirmatory tests reported in Table 1 also eliminated the presence of hemoglobinopathy and G6PD deficiency. Peripheral smear examination demonstrated in Figure 1 was not a characteristic of spherocytosis or elliptocytosis. There are various causes of acquired hemolytic anemia including but not limited to autoimmune, drug-induced, microangiopathic hemolytic anemia, paroxysmal nocturnal hemoglobinuria (PNH), infections, chemicals, nutritional deficiency such as vitamin B12 and folate deficiency, sever burn, and radiation. Due to multiple etiologies, numbers of workup were performed for this patient to rule out different causes of acquired hemolytic anemia. The possibility of hemolytic anemia secondary to severe burn or radiation exposure was eliminated since the patient denied such history. His Coombs test, cold agglutinin test, ANA, and anti-phospholipid antibodies were negative which argue against autoimmune hemolytic anemia. Drug-induced or chemical-related hemolysis was also less likely since the patient reported taking only Metamucil and he has no history of chemical-related exposure. Furthermore, since the patient reported recent viral-like illness, numbers of tests were performed to rule out infectious causes which were negative (see Table 1). Microangiopathic hemolytic anemia, such as thrombotic thrombocytopenic purpura (TTP), hemolytic uremic syndrome (HUS), and disseminated intravascular coagulation (DIC), is also less likely since the patient has normal platelet count, no renal or neurological abnormality, normal ADAMTS13 activity, and normal coagulation. Anther intravascular hemolysis such as valvular heart disease was also excluded since he does not have murmur on physical examination and prior history of cardiac disease. In addition, negative CD55/59 lessens the suspicion of PNH. The likely cause of hemolytic anemia in this case was due to vitamin B12 deficiency since serum B12 was extremely low and the diagnosis was confirmed when homocysteine and MMA levels were elevated.

Commonly, vitamin B12 deficiency is associated with macrocytic anemia. However, the patient's mean corpuscular volume (MCV) was normal which suggested the presence of concomitant iron deficiency anemia. Increased RDW was consistent with poikilocytosis and anisocytosis picture in the peripheral blood smear. Also, low reticulocyte index (<2) indicates defective erythropoiesis which can be explained by severe vitamin B12 deficiency and iron deficiency anemia. Low serum ferritin, iron level, and transferrin saturation (TSAT) confirmed the diagnosis.

Vitamin B12 or cobalamin deficiency is common in elderly population [2]. The prevalence in general population is around 20% in industrialized nations, although it can range from 5% to 80% depending on definition and study population [2, 3]. The main source of vitamin B12 is animal product such as meat, milk, egg, fish, and shellfish [4]. Certain plant such as blue-green algae contains large amount of vitamin B12, but the compound was found to be inactive in mammals [4]. Hence, strict vegetarians have a greater risk of developing vitamin B12 deficiency [4].

Vitamin B12 functions as a cofactor or coenzyme that participates in various biochemical reactions, including DNA synthesis [2], which promotes normal maturation of blood cells. It also contributes to the myelination of central nervous system as well as maintenance of its function [1]. The deficiency of vitamin B12 can lead to megaloblastic anemia and neuropathy [1, 4, 5]. Intramedullary destruction or hemolysis of fragile and abnormal red blood cell precursors is the result of ineffective erythropoiesis secondary to defective DNA and cell maturation [1, 5]. The hemolytic picture may resemble microangiopathic hemolytic anemia [1]. If such condition persisted, it may deplete iron storage and lead to concomitant iron deficiency anemia [5]. 

Pernicious anemia is the most common cause of cobalamin deficiency worldwide [1]. However, among elderly population, food-cobalamin malabsorption which is caused by gradual atrophy of gastric mucosa and hypochlorhydria is responsible for the majority of cases [2, 3, 6]. The syndrome is characterized by the inability to release cobalamin from food for absorption due to reduced gastric acid secretion, but unbound cobalamin can be absorbed normally [2, 3].

The diagnosis of vitamin B12 deficiency can be done with initial testing of vitamin B12 assay [1]. Extremely low level (<100 pg per milliliter) is usually associated with clinical deficiency [1]. The disease is confirmed when both MMA and homocysteine levels are elevated [1]. Both MMA and homocysteine levels can be used to document adequate therapy as well since both levels will decrease with vitamin B12 supplement [1]. Folate deficiency can also cause serum homocysteine to be elevated but not MMA [1]. Thus, coexisting folate deficiency should be sought in a patient with suspected vitamin B12 deficiency.

In our patient, after the diagnoses of vitamin B12 deficiency and iron deficiency anemia were confirmed, further investigations were pursued to explain the cause. Celiac disease antibodies and intrinsic factor antibody were found to be negative which reduced the possibility of celiac disease and pernicious anemia. Since the patient never had history of abdominal pain, indigestion, acid reflux symptoms, or use of acid reducing medication, it is less likely that atrophic gastritis or *Helicobacter pylori* infection is the contributing cause, although EGD evaluation with pathological biopsy would be helpful in this case. The Schilling test was not performed since it is not available in our institution. As for the cause of his iron deficiency, gastrointestinal bleeding is a less likely explanation since stool hemoccult test was negative. It is probable that severe intramedullary destruction and hemolysis of erythrocytes from ineffective erythropoiesis result in depletion of iron storage and lead to iron deficiency anemia. Although anemia from epistaxis is possible, the bleeding is too intermittent and negligible to allow for iron deficiency. Based on thorough historical review, it is likely that the cause of his vitamin B12 deficiency was due to nutritional insufficiency. Decrease in dietary iron can further precipitate his iron deficiency anemia. As discussed, since his diet mainly consists of vegetable and legumes, it is doubtful that folate deficiency coexisted. This hypothesis was verified by normal folate RBC level. The metabolism of folate and B12 is essentially linked. In the absence of vitamin B12, folate is trapped as N5-methyltetrahydrofolate, and therefore cannot be reutilized [7]. Hence, a patient who has low RBC folate may indicate primary vitamin B12 deficiency [7].

The treatment of cobalamin deficiency required replacement of vitamin B12. Daily high dose oral therapy (1000 to 2000 mcg per day) is as effective as parenteral formula in several randomized studies [1]. Oral therapy can also be used in patients with pernicious anemia, atrophic gastritis, or a history of gastric surgery or ileal resection [1]. Our patient was initially treated with intramuscular injection of vitamin B12 together with iron and folate supplement which showed significant improvement in symptoms in 1 week. Four weeks after discharge, his Hb and red cell indices continue to improve with complete resolution of hemolysis. Vitamin B12 level and RBC folate have normalized. Iron study and serum ferritin level also improved.

This case displayed the complexity of vitamin B12 deficiency where clinicians should be familiar. Once the diagnosis is confirmed, further investigation is warranted to explain the etiology. Life-long therapy is necessary in disease such as pernicious anemia and malabsorptive conditions. Individuals who are on strict vegetarian diet should be advised to take supplement as recommended by national guidelines in order to prevent harmful hematologic and neurological sequelae.

## Figures and Tables

**Table 1:** Laboratory results on admission and during followup.

Lab	Reference	Admission	4 weeks
WBC (/mm^3^)	4.2–11.0 k/mm cu	4.7	5.9
Hb (g/dL)	13.5–17.0 g/dL	5.9	10.1
Hct (%)	41.0–52.0%	18.6	32.3
Plt (/mm^3^)	140–400 k/mm cu	161	241
MCV (fL)	80.0–100.0 fL	87.1	85.8
MCH (pg/red cell)	26.0–33.0 pg	27.4	26.9
MCHC (g/dL)	32.0–37.0%	31.4	31.4
RDW (%)	11.0–14.5%	30.7	21.6
Differential count (%)			
Neutrophil	40.0–72.0%	59.3	53.2
Eosinophil	0.0–10.0%	1.1	1.4
Monocyte	4.0–12.0%	4.2	7.8
Lymphocyte	17.0–45.0%	35.1	36.6
Reticulocytes (%)	0.5–2.8%	2.4	1.2
Reticulocyte index	1.0–2.0%	0.4	0.6
PT (sec)/INR	8.9–11.9 sec/0.9–1.1	15.2/1.4	11.9/1.1
PTT (sec)	23–33 sec	27	NA
Total/direct bilirubin	0.0–1.0 mg/dL/0.0–0.3 mg/dL	3.7/0.4	1.4/NA
LDH (U/L)	135–225 IU/L	701	189
Haptoglobin (mg/dL)	36–195 mg/dL	<6	98
Vitamin B12 (pg/mL)	211–946 pg/mL	<30	>2000
RBC folate (ng/mL)	>280 NG/ML RBC	697	591
Homocysteine (Umol/L)	3.7–13.9 Umol/L	100	NA
Methylmalonic acid (nmol/L)	87–318 nmol/L	22708	NA
Iron (ug/dL)	45–160 ug/dL	36	107
Ferritin (ng/mL)	30–400 ng/mL	7	19
Iron saturation (%)	20–55%	9	23
TIBC (ug/dL)	228.0–428.0 ug/dL	403	456
IgA	50–400 mg/dL	612	NA
TTG IgA	<20.0 Units	10.8	NA
Endomysial Ab	Negative	Negative	NA
Intrinsic factor Ab	Negative	Negative	NA
